# Arabidopsis AtERF014 acts as a dual regulator that differentially modulates immunity against *Pseudomonas syringae* pv. *tomato* and *Botrytis cinerea*

**DOI:** 10.1038/srep30251

**Published:** 2016-07-22

**Authors:** Huijuan Zhang, Yongbo Hong, Lei Huang, Dayong Li, Fengming Song

**Affiliations:** 1 National Key Laboratory for Rice Biology, Institute of Biotechnology, Zhejiang University, Hangzhou 310058, P.R. China

## Abstract

ERF transcription factors play critical roles in plant immune responses. Here, we report the function of AtERF014, a nucleus-localized transcriptional activator, in Arabidopsis immunity. Expression of *AtERF014* was induced by *Pseudomonas syringae* pv*. tomato* (*Pst*) and *Botrytis cinerea* (*Bc*). AtERF014-overexpressing (OE) plants displayed increased *Pst* resistance but decreased *Bc* resistance, whereas AtERF014-RNAi plants exhibited decreased *Pst* resistance but increased *Bc* resistance. After *Pst* infection, expression of salicylic acid (SA)-responsive genes *AtPR1* and *AtPR5* in AtERF014-OE plants and of a jasmonic acid/ethylene-responsive gene *AtPDF1.2* in AtERF014-RNAi plants was intensified but expression of *AtPDF1.2* in AtERF014-OE plants and of *AtPR1* and *AtPR5* in AtERF014-RNAi plants was weakened. After *Bc* infection, expression of *AtPR1* and *AtPR5* in AtERF014-OE plants was attenuated but expression of *AtPR1*, *AtPR5* and *AtPDF1.2* in AtERF014-RNAi plants was strengthened. Pathogen- and flg22-induced ROS burst, expression of PTI genes and SA-induced defense were partially suppressed in AtERF014-RNAi plants, whereas pathogen-induced ROS and flg22-induced immune response were strengthened in AtER014-OE plants. Altered expression of *AtERR014* affected expression of pectin biosynthetic genes and pectin content in AtERF014-RNAi plants was decreased. These data demonstrate that AtERF014 acts as a dual regulator that differentially modulates immunity against *Pst* and *Bc* in Arabidopsis.

Plants are frequently exposed to attack by potential microbes during their lifespan and thus they have developed to possess arrays of complicated molecular mechanisms to cope with the invading pathogens. The plant innate immunity system comprises of two layers of immune responses, called pathogen-associated molecular pattern (PAMP)-triggered immunity (PTI) and effector-triggered immunity (ETI)^1^,^2^. PTI and ETI are activated upon recognition of PAMPs such as flagellin, EF-Tu and chitin^3^,^4^,^5^,^6^ and pathogen-derived specific effectors^7^,^8^ by pattern-recognition receptors or specified R proteins in plants, respectively. In addition, plants have also developed to possess several forms of inducible immunity, e.g. systemic acquired resistance and induced systemic resistance, which becomes activated upon pathogen infection or treatment of elicitors^9^,^10^.

Upon perception of pathogen-derived signals, plants often activate a network of defense hormone-mediated signaling pathways^11^, which ultimately lead to transcriptional reprogramming that coordinately regulates expression of a large set of genes. For example, one-third of the Arabidopsis genome changes in expression during the first 48 hr after infection by *Botrytis cinerea*^12^, whereas approximately 4000 genes are differentially expressed in immune response induced by *Pseudomonas syringae* pv. *tomato* DC3000*hrpA*^13^. Such large-scale transcriptional reprogramming of gene expression in a specific immune response obviously requires a concerted function of different types of transcription factors (TFs) in both temporal and spatial manners. Recent genetic studies have demonstrated that a number of TFs from the families of WRKY, AP2/ERF (Apetala2/ethylene responsive factor) and NAC play crucial roles in immune responses against pathogens^14^,^15^,^16^,^17^.

The AP2/ERF superfamily is a large plant-specific TF family^18^ and is characterized by the presence of one or two AP2/ERF domains that consist of 58 or 59 conserved amino acid residues^19^. The AP2/ERF superfamily can be divided into three subfamilies, namely AP2, RAV and ERF, and 122 out of 147 members in Arabidopsis are ERF TFs^18^,^20^,^21^. The ERFs can act as transcription activators or repressors; for example, Arabidopsis AtERF1, AtERF2 and AtERF5 are activators while AtERF3, AtERF4 and AtERF7 are repressors^22^. Generally, ERF TFs regulate the expression of genes, whose promoters harbor at least one core GCC box, and most of these GCC box-containing genes are defense-related^19^,^23^,^24^,^25^.

ERF TFs are heavily linked with the regulation of immune response in plants and are integrators of different defense hormone pathways^16^,^17^,^20^,^23^. In Arabidopsis, a total of 53 AP2/ERF TFs are differentially expressed following *B. cinerea* infection^12^. Functional studies using knockout/knockdown mutants have revealed that 10 out of 17 members in the group IX of the ERF family, including AtERF92 (AtERF1), AtERF93 (AtERF15), AtERF94 (ORA59), AtERF96, AtERF97 (AtERF14), AtERF100 (AtERF-1), AtERF101 (AtERF2), AtERF102 (AtERF5), AtERF103 (AtERF6) and AtERF104, play important roles in regulating immune response against pathogens including *B. cinerea*^25^,^26^,^27^,^28^,^29^,^30^,^31^,^32^,^33^,^34^,^35^,^36^,^37^. In addition, AtERF078 (AtERF4) and AtERF080 (AtERF9), belonging to group VIII, act as negative regulators of resistance to *Fusarium oxysporum* and *B. cinerea*, respectively^29^,^38^; whereas AtERF075 (AtRAP2.2), a member of group VII, functions as an important regulator in resistance to *B. cinerea*^39^. In addition, overexpression of *AtERF11* (*AtDEAR1*), a member of group II, resulted in lesion-like cell death, elevated SA level, constitutive expression of defense genes and increased resistance to *P. syringae* pv. *tomato* DC3000^40^. Most of the reported ERF TFs, including AtERF1, AtERF5, AtERF6, AtERF14, ORA59 and AtERF96, function in Arabidopsis immune response through modulating jasmonic acid (JA)/ethylene (ET)-mediated signaling pathway, resulting in expression of defense genes including *AtPDF1.2*^27^,^30^,^31^,^32^,^36^,^38^,^41^,^42^. It was also found that AtERF5 and AtERF15 are involved in PTI and positively regulate salicylic acid (SA)-mediated signaling pathway that is involved in resistance to *P. syringae* pv*. tomato* DC3000^33^,^35^,^40^. Collectively, these data strongly demonstrate the importance of ERF TFs in regulation of Arabidopsis immune responses through modification of different defense signaling pathways.

AtERF014, a member of group II in the ERF family^21^, was recently reported to be involved in pectin biosynthesis^43^. However, the biological function of AtERF014 remains elusive. The present study focused on the function of AtEFR014 in disease resistance to *P. syringae* pv. *tomato* DC3000 and *B. cinerea*. Functional analyses using AtERF014-OE and AtERF014-RNAi lines suggest that AtERF014 acts as a positive regulator of resistance against *Pst* DC3000 but functions as a negative regulator of resistance to *B. cinerea*. Furthermore, AtERF014 is involved in flg22-induced PTI response and SA-induced defense response. Our data demonstrate that AtERF014 is a dual regulator that differentially modulates immunity against *P. syringae* pv. *tomato* DC3000 and *B. cinerea* in Arabidopsis.

## Results

### *AtERF014* is responsive to pathogen infection and defense signaling hormones

**Figure 1:**
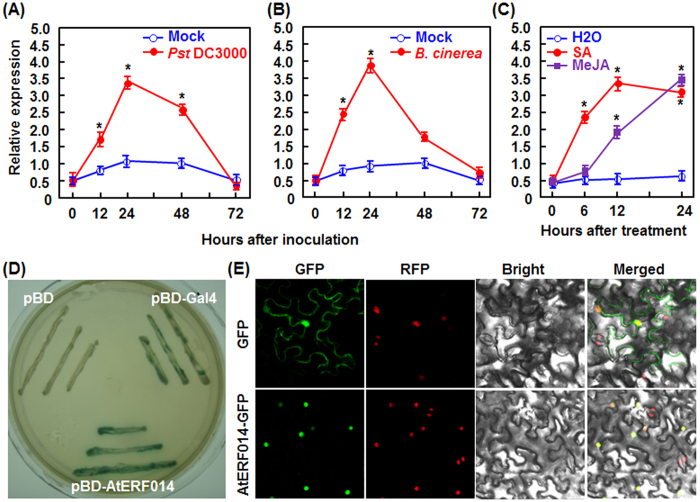
Pathogen-induced expression of *AtERF014* and biochemical characteristics of AtERF014. (**A**–**C**) Expression patterns of *AtERF014* induced by *P. syringae* pv. *tomato* DC3000 (**A**) and *B. cinerea* (**B**) or by defense signaling hormones (**C**). Four-week-old plants were inoculated with *Pst* DC3000, *B. cinerea* spore suspension, or similar volume of buffer as mock controls (**A**,**B**). The Arabidopsis plants were treated by foliar spraying with solutions of 1 mM SA or 100 μM MeJA (**C**). The transcript level of *AtERF014* was analyzed and relative expression is shown as folds of the transcript level of the internal *AtActin* gene. Data presented are the means ± SD from three independent experiments and * indicates significant difference at *p* < 0.05 level between the inoculated/treated plants and mock control plants. (**D**) AtERF014 is a transcription activator. Yeasts harboring pBD-AtERF014, pBD empty vector (a negative control) and pBD-GAL4 (a positive control) were grown on SD/Trp^−^ medium and β-galactosidase activity was examined by addition of X-α-gal. (**E**) AtERF014 is localized in nucleus. Leaves of *Nicotinana benthamiana* plants were collected at 24 hr after infiltration of agrobacteria and photos were taken in dark field for GFP and RFP, bright field for cell morphology and in combination (merged), respectively.

To explore the involvement in disease resistance, we examined whether expression of *AtERF014* could be induced by pathogen infection and defense signaling hormones such as SA and JA. As shown in Fig. 1A, the transcript level of *AtERF014* increased as early as 12 hr post-inoculation (hpi), peaked with ~6 folds at 24 hpi, maintained at relatively higher level at 48 hpi and then decreased to basal level at 72 hpi after infection by *Pst* DC3000. Similar kinetics of change in the transcript level of *AtERF014* was observed in *B. cinerea*-infected plants, showing a peak of 7 folds at 24 hpi (Fig. 1B). In addition, the expression of *AtERF014* was also induced by SA and MeJA but showed different patterns. In SA-treated plants, the transcript level of *AtERF014* increased rapidly at 3 hr post-treatment (hpt), peaked with 6 folds at 12 hpt and maintained at relatively higher level until 24 hpt (Fig. 1C). By contrast, the transcript level of *AtERF014* in methyl jasmonate (MeJA)-treated plants increased gradually and peaked with 6 folds at 24 hpt (Fig. 1C). These data suggest that *AtERF014* is responsive to pathogen infection and defense signaling hormones.

### AtERF014 is a transcriptional activator that is localized in nucleus

The biochemical characters of AtERF014 protein were examined by analyzing the transactivation activity in yeast and subcellular localization *in planta*. In transactivation activity assay, yeasts carrying pBD-AtERF014, pBD-GAL4 (a positive control) or pBD empty vector (a negative control) grew on SD/Trp^–^ medium but only yeasts carrying pBD-AtERF014 or pBD-GAL4 exhibited β-galactosidase activity, as revealed by the production of blue pigment after addition of X-α-gal (Fig. 1D). In subcellular localization assay, the GFP alone was seen ubiquitously in cells without specific compartmental localization, whereas the GFP::AtERF014 fusion was solely localized in nucleus, co-localized with the known nucleus marker RFP–H2B protein^44^ (Fig. 1E). These results indicate that AtERF014 may be a transcriptional activator that is localized in nucleus.

### Altered expression of *AtERF014* does not affect the growth and development in AtERF014-OE and AtERF014-RNAi plants

**Figure 2:**
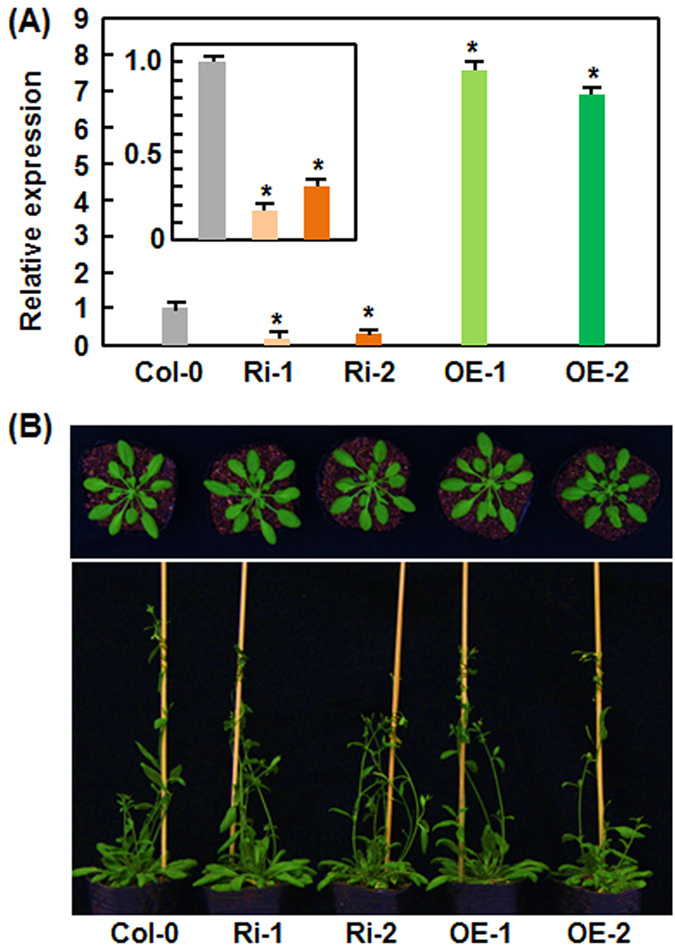
Altered expression of *AtERF014* does not affect the growth and development in AtERF014-OE and AtERF014-RNAi plants. (**A**) Transcript levels of *AtERF014* in AtERF014-OE and AtERF014-RNAi lines. Transcript levels of *AtERF014* in AtERF014-OE and AtERF014-RNAi plants were shown as folds of that in WT, which was as 1. Data presented are the means ± SD from three independent experiments and * indicates significant difference at *p* < 0.05 level between the AtERF014-OE/AtERF014-RNAi and WT plants. (**B**) Morphological and developmental phenotypes of AtERF014-OE and AtERF014-RNAi plants at vegetable (upper panel) and reproductive stages (lower panel). Upper panel, 4-week-old plants; lower panel, 6-week-old plants.

To better understand the biological function of *AtERF014*, we generated transgenic lines with overexpression or RNA interfering (RNAi)-mediated suppression of *AtERF014*. Two homozygous single-copy AtERF014-OE and AtERF014-RNAi lines (T3 generation) were chosen for further studies. The transcript levels of *AtERF014* in AtERF014-OE lines were 6.56 and 5.84 times higher than that in WT while the transcript levels in AtERF014-RNAi lines were 18% and 29% of that in WT (Fig. 2A). The AtERF014-OE and AtERF014-RNAi plants grew and developed normally, indistinguishable from WT, at vegetable and reproductive stages (Fig. 2B). Thus, it is likely that altered expression of *AtERF014* does not affect the growth and development in AtERF014-OE and AtERF014-RNAi plants, implying a limited role for *AtERF014* in growth and development.

### Altered expression of *AtERF014* affects the basal immunity in AtERF014-OE and AtERF014-RNAi plants against *Pst* DC3000

**Figure 3:**
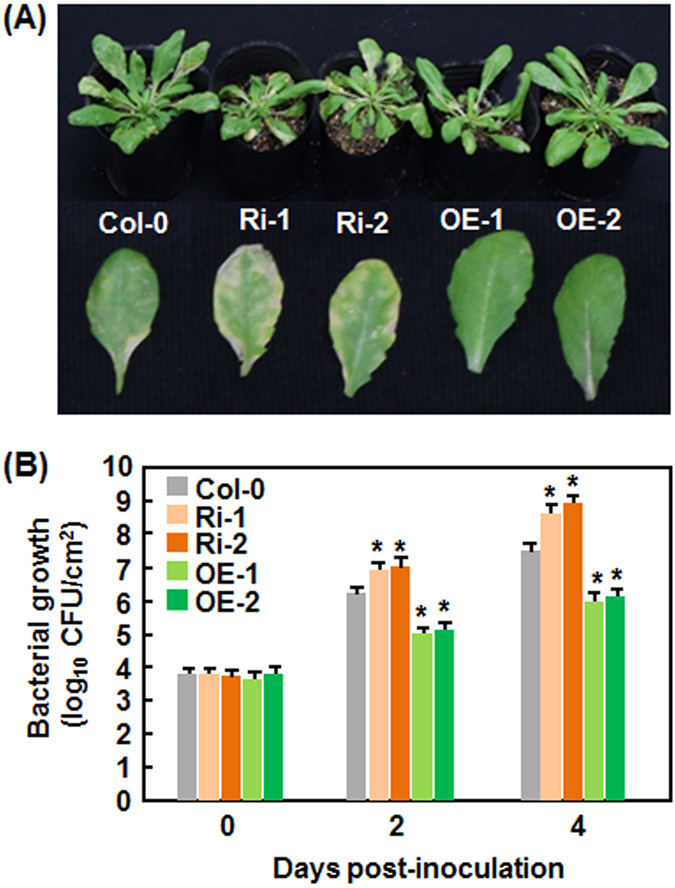
Altered disease resistance of AtERF014-OE and AtERF014-RNAi plants against *P. syringae* pv. *tomato* DC3000. (**A**) Typical *P. syringae* pv. *tomato* DC3000-provoked disease on WT, AtERF014-OE and AtERF014-RNAi plants. Four-week-old plants were inoculated with *Pst* DC3000 and photos were taken at 4 dpi. (**B**) *In planta* bacterial growth in inoculated leaves. Data presented are the means ± standard deviation from three independent experiments and * above the columns indicate significant differences at *p* < 0.05 level between AtERF014-OE/AtERF014-RNAi and WT plants.

Having shown that the expression of *AtERF014* was induced by *Pst* DC3000, we first examined whether altered expression of *AtERF014* affected the resistance of AtERF014-OE and AtERF014-RNAi plants to this bacterial pathogen. At 4 days post-inoculation (dpi), typical *Pst* DC3000-provoked disease with chlorotic lesion was seen in WT plants (Fig. 3A). At the same time, AtERF014-RNAi plants displayed much severe disease with extensive chlorotic lesion while AtERF014-OE plants showed less severe disease (Fig. 3A). Accordingly, bacterial populations in AtERF014-RNAi plants were 7 and 21 folds higher while the populations in AtERF014-OE plants were 13 and 32 times lower, as compared to those in WT at 2 and 4 dpi, respectively (Fig. 3B). These data indicate that overexpression of *AtERF014* leads to an increased resistance while suppression of *AtERF014* results in a decreased resistance against *Pst* DC3000.

### Altered expression of *AtERF014* affects the basal immunity in AtERF014-OE and AtERF014-RNAi plants against *B. cinerea*

**Figure 4:**
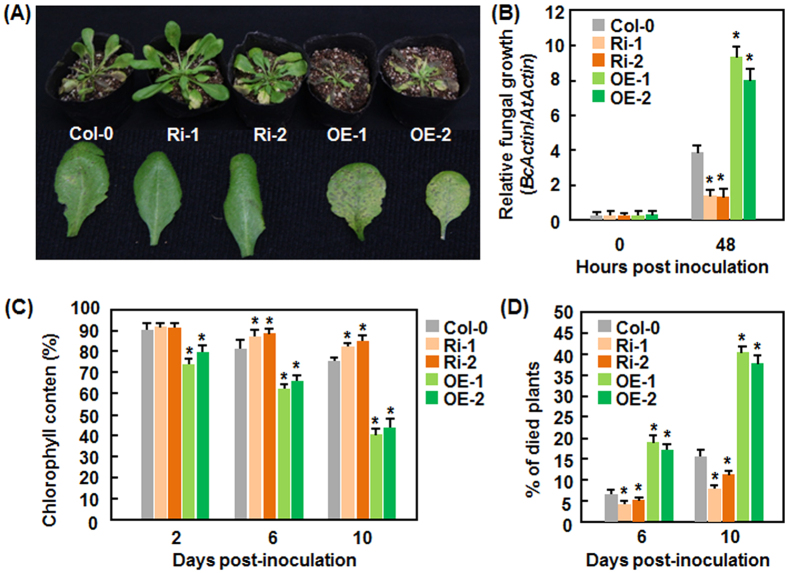
Altered disease resistance of AtERF014-OE and AtERF014-RNAi plants against *B. cinerea*. (**A**) Typical *B. cinerea*-provoked disease on WT, AtERF014-OE and AtERF014-RNAi plants. Four-week-old plants were inoculated by foliar spraying with *B. cinerea* spore suspension and photos were taken at 5 dpi. (**B**) *In planta* fungal growth in inoculated plants. Fungal growth was shown as ratios of the transcript level of *B. cinerea BcActinA* to the transcript level of Arabidopsis *AtActin*. (**C**) Changes in chlorophyll contents in leaves of inoculated plants. The chlorophyll contents in *B. cinerea*-infected plants were shown as percentages of those in corresponding mock-inoculated plants. (**D**) Percentages of died WT, AtERF014-OE and AtERF014-RNAi plants after *B. cinerea* infection. Data presented are the means ± standard deviation from three independent experiments and * above the columns indicate significant differences at *p* < 0.05 level between AtERF014-OE/AtERF014-RNAi and WT plants.

We next assessed whether altered expression of *AtERF014* affected the resistance of AtERF014-OE and AtERF014-RNAi plants to *B. cinerea*, a necrotrophic fungus causing grey mold disease. After infection, typical *B. cinerea*-provoked disease symptom was seen in WT at 4 dpi (Fig. 4A). More severe disease symptoms were observed on leaves of AtERF014-OE plants but less disease was seen on leaves of AtERF014-RNAi plants, compared with that in WT (Fig. 4A). Similarly, the AtERF014-OE plants supported greater growth of *B. cinerea*, showing 1.4 and 1.1 times higher, while the AtERF014-RNAi plants supported lower growth, resulting in 60% of reduction, as compared with that in WT, at 48 hpi, respectively (Fig. 4B). Chlorophyll content in AtERF014-OE plants decreased gradually with disease development and exhibited 30–35% lower while chlorophyll content in AtERF014-RNAi plants decreased slowly and showed 6–9% higher, than that in WT, at 10 dpi (Fig. 4C). Furthermore, more AtERF014-OE plants died (38–41% of death) while more AtERF014-RNAi plants survived (7–11% of death), as compared to that in WT (16% of death) (Fig. 4D). These results indicate overexpression of *AtERF014* weakens while suppression of *AtERF014* strengthens the resistance against *B. cinerea*.

### Altered expression of *AtERF014* affects *Pst* DC3000- and *B. cinerea*-induced defense response in AtERF014-OE and AtERF014-RNAi plants

**Figure 5:**
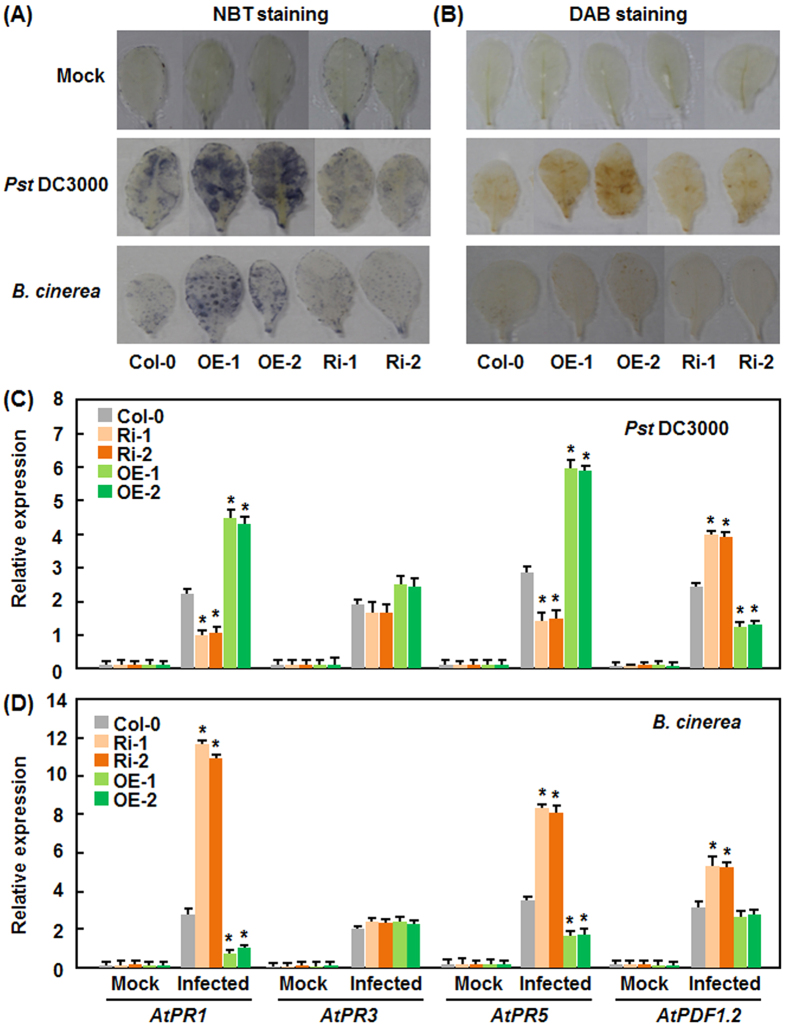
Altered pathogen-induced accumulation of ROS accumulation and expression of defense genes in AtERF014-OE and AtERF014-RNAi plants after infection with *P. syringae* pv. *tomato* DC3000 or *B. cinerea*. Four-week-old plants were inoculated with *Pst* DC3000, *B. cinerea* spore suspension or similar volume of solutions as mock controls. Leaf samples were collected at 24 hr after inoculation. (**A**,**B**) *In situ* detection of superoxide anion (**A**) and H_2_O_2_ (**B**) accumulation in leaves of inoculated plants, detected by NBT or DAB staining, respectively. (**C**,**D**) Changes in expression of defense genes in AtERF014-OE and AtERF014-RNAi plants after infection with *Pst* DC3000 (**C**) or *B. cinerea* (**D**). Data presented are the means ± standard deviation from three independent experiments and * above the columns indicate significant differences at *p* < 0.05 level between AtERF014-OE/AtERF014-RNAi and WT plants.

To examine whether altered expression of *AtERF014* affected the defense response, we analyzed and compared the patterns for accumulation of reactive oxygen species (ROS) and expression of defense genes among WT, AtERF014-OE and AtERF014-RNAi plants after infection by *Pst* DC3000 or *B. cinerea*. In *in situ* detection of ROS assays, accumulation of superoxide anion and H_2_O_2_ in leaves was detected by nitroblue tetrazolium (NBT)^45^ and 3, 3-diaminobenzidine (DAB) staining^46^,^47^, respectively. Without pathogen challenge, no difference in accumulation of superoxide anion and H_2_O_2_ was seen among WT, AtERF014-OE and AtERF014-RNAi plants (Fig. 5A,B). At 24 hr after infection by *Pst* DC3000 or *B. cinerea*, accumulation of superoxide anion and H_2_O_2_ in inoculated leaves increased obviously except the case of H_2_O_2_ in *B. cinerea*-infected leaves (Fig. 5A,B). However, more staining for superoxide anion and H_2_O_2_ in AtERF014-OE leaves while less in AtERF014-RNAi leaves was observed, as compared to that in WT (Fig. 5A,B). On the other hand, the expression levels of some selected defense genes including *AtPR1*, *AtPR3*, *AtPR5* and *AtPDF1.2* in AtERF014-OE and AtERF014-RNAi plants were comparable to those in WT without infection with *Pst* DC3000 or *B. cinerea* (Fig. 5C,D). As compared with those in WT, higher expression levels of *AtPR1* and *AtPR5* and a lowered expression level of *AtPDF1.2* in AtERF014-OE plants were observed, whereas lowered expression levels of *AtPR1* and *AtPR5* and a higher expression level of *AtPDF1.2* in AtERF014-RNAi plants were detected, at 24 hr after infection by *Pst* DC3000 (Fig. 5C,D). By contrast, lowered expression levels of *AtPR1* and *AtPR5* and a higher expression level of *AtPDF1.2* in AtERF014-OE plants were seen, while higher expression levels of *AtPR1* and *AtPR5* and a lowered expression level of *AtPDF1.2* in AtERF014-RNAi plants were observed, as compared to those in WT at 24 hr after infection with *B. cinerea* (Fig. 5C,D). There was no difference in pathogen-induced expression of *AtPR3* among WT, AtERF014-OE and AtERF014-RNAi plants (Fig. 5C,D). Taken together, these data suggest that altered expression of *AtERF014* affects the pathogen-induced defense responses including ROS accumulation and expression of defense genes in AtERF014-OE and AtERF014-RNAi plants.

### Altered expression of *AtERF014* affects flg22-triggered immune response in AtERF014-OE and AtERF014-RNAi plants

**Figure 6:**
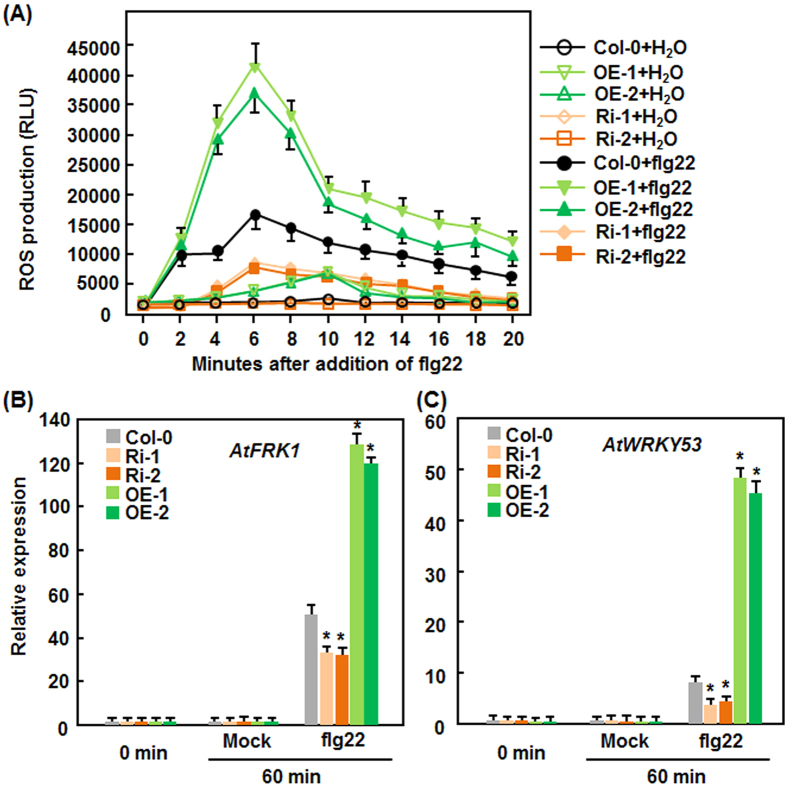
Altered flg22-triggered immune responses in AtERF014-OE and AtERF014-RNAi plants. (**A**) flg22-induced ROS burst. ROS burst was monitored immediately after addition of flg22 (100 nM). (**B,C**) flg22-induced expression of the PTI marker genes AtFRK1 (**B**) and AtWRKY53 (**C**). Data presented are the means ± standard deviation from three independent experiments and * above the columns indicate significant differences at *p* < 0.05 level between AtERF014-OE/AtERF014-RNAi and WT plants.

To examine whether AtERF014 has a function in Arabidopsis PTI response, we compared the occurrence of flg22-induced ROS burst and flg22-induced expression of PTI marker genes among WT, AtERF014-OE and AtERF014-RNAi plants. In ROS burst assay, no significant ROS burst was observed in WT, AtERF014-OE and AtERF014-RNAi leaves without treatment of flg22 (Fig. 6A). Compared with a significant flg22-induced ROS burst in WT leaves at 6 min, much enhanced flg22-induced ROS bursts were seen in AtERF014-OE leaves while only a much reduced ROS burst was detected in AtERF014-RNAi leaves after addition of flg22 (Fig. 6A). Similarly, the expression levels of *FLG22-INDUCED RECEPTOR-LIKE KINASE 1* (*AtFRK1*) and *AtWRKY53*, two well-known PTI-responsive genes^48^,^49^, in AtERF014-OE and AtERF014-RNAi leaves were comparable to those in WT without flg22 treatment or at 0 hr after addition of flg22 (Fig. 6B,C), suggesting that altered expression of *AtERF014* did not affect the expression of these two PTI genes. At 60 min after treatment, flg22 significantly upregulated the expression of *AtFRK1* and *AtWRKY53* in WT, AtERF014-OE and AtERF014-RNAi leaves with distinct patterns (Fig. 6B,C). As compared with the levels in flg22-treated WT leaves, the flg22-upregulated expression of *AtFRK1* and *AtWRKY53* in AtERF014-OE leaves was further increased, resulting in 1.4–1.6 times and 4–5 times of increases, whereas the expression of these two genes in AtERF014-RNAi leaves was markedly suppressed, leading to 25–30% and 50–60% of reduction (Fig. 6A,B). These data indicate that overexpression of *AtERF014* strengthens while suppression of *AtERF014* attenuates the flg22-induced PTI response, implying a function for AtERF014 in PTI response in Arabidopsis.

### Suppression of *AtERF014* attenuates the SA-induced defense response in AtERF014-RNAi plants

**Figure 7:**
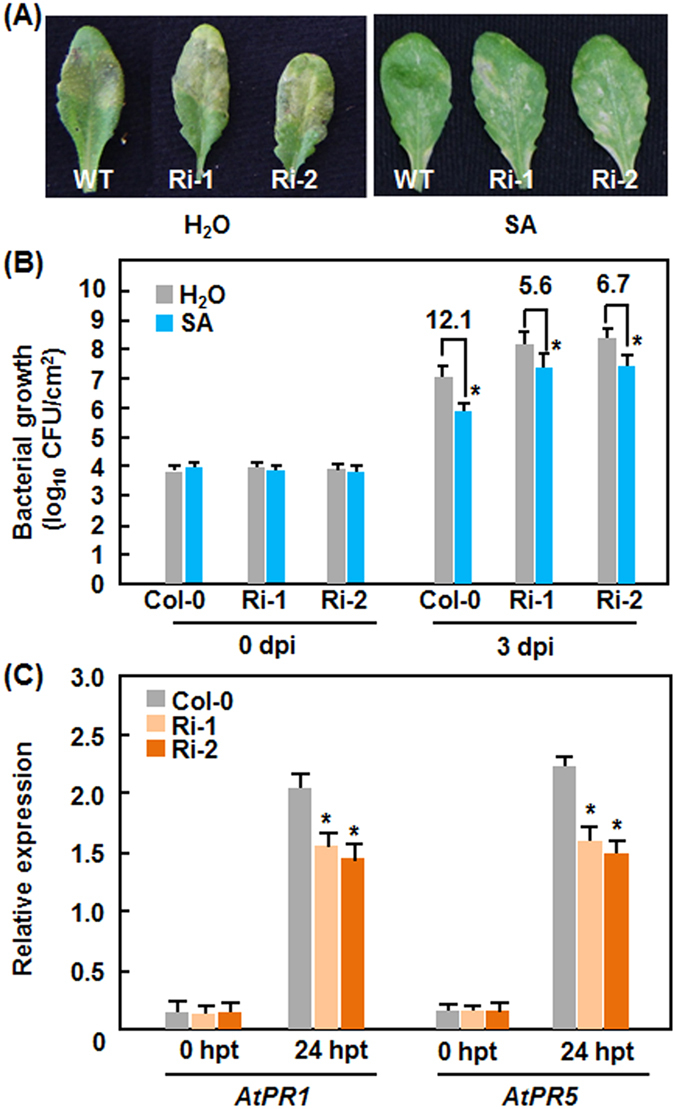
Attenuated SA-induced defense response in AtERF014-RNAi plants. (**A**) and (**B**) SA-induced resistance against *P. syringae* pv. *tomato* DC3000 was attenuated in AtERF014-RNAi plants. Four-week-old plants were treated with 1 mM SA or water and then inoculated with *Pst* DC3000 at 24 hr after SA treatment. (**A**) *P. syringae* pv. *tomato* DC3000-provoked disease in leaves at 4 dpi and (**B**) *in planta* bacterial growth in inoculated leaves. (**C**) SA-induced expression of defense genes was partially suppressed in AtERF014-RNAi plants. Relative expression was shown as folds of the transcript level of an internal *AtActin* gene. Data presented are the means ± standard deviation from three independent experiments and * above the columns indicate significant differences at *p* < 0.05 level between AtERF014-RNAi and WT plants. dpi, days post-inoculation; hpt, hour post-treatment.

To explore whether *AtERF014* is required for SA-induced defense response, we evaluated the SA-induced resistance between AtERF014-RNAi and WT plants by comparing disease phenotype and bacterial population in water- and SA-treated plants after inoculation with *Pst* DC3000. SA-treated WT plants showed less disease than water-treated plants at 4 dpi (Fig. 7A). Disease in SA-treated AtERF014-RNAi plants was less than that in water-treated plants but was much severe than that in SA-treated WT plants (Fig. 7A). Accordingly, *in planta* growth of *Pst* DC3000 in inoculated leaves was consistent with the disease severity observed. At 0 dpi, no significant difference in bacterial growth was seen between AtERF014-RNAi and WT plants treated with or without 1 mM SA. At 3 dpi, the bacterial titers in inoculated leaves of water-treated WT, AtERF014-RNAi-1 and AtERF014-RNAi-2 plants increased to 1.10 × 10^7^ CFU/cm^2^, 1.12 × 10^8^ CFU/cm^2^ and 2.04 × 10^8^ CFU/cm^2^, respectively, whereas the titers in inoculated leaves of SA-treated WT, AtERF014-RNAi-1 and AtERF014-RNAi-2 plants were 8.51 × 10^5^ CFU/cm^2^, 2.29 × 10^7^ CFU/cm^2^ and 2.88 × 10^7^ CFU/cm^2^, respectively, showing 12.1, 5.6 and 6.7 times lower than those in corresponding water-treated plants (Fig. 7B). The expression levels of *AtPR1* and *AtPR5* in AtERF014-RNAi plants were comparable to those in WT without SA treatment; their expression was significantly induced by SA in both AtERF014-RNAi and WT plants (Fig. 7C). However, the SA-induced expression of *AtPR1* and *AtPR5* in AtERF014-RNAi plants was weakened as compared to those in WT (Fig. 7C). These results indicate that suppression of *AtERF014* weakens the SA-induced defense response in AtERF014-RNAi plants.

### Altered expression of *AtERF014* affects the pectin content and expression of pectin biosynthetic genes in AtERF014-OE and AtERF014-RNAi plants

**Figure 8:**
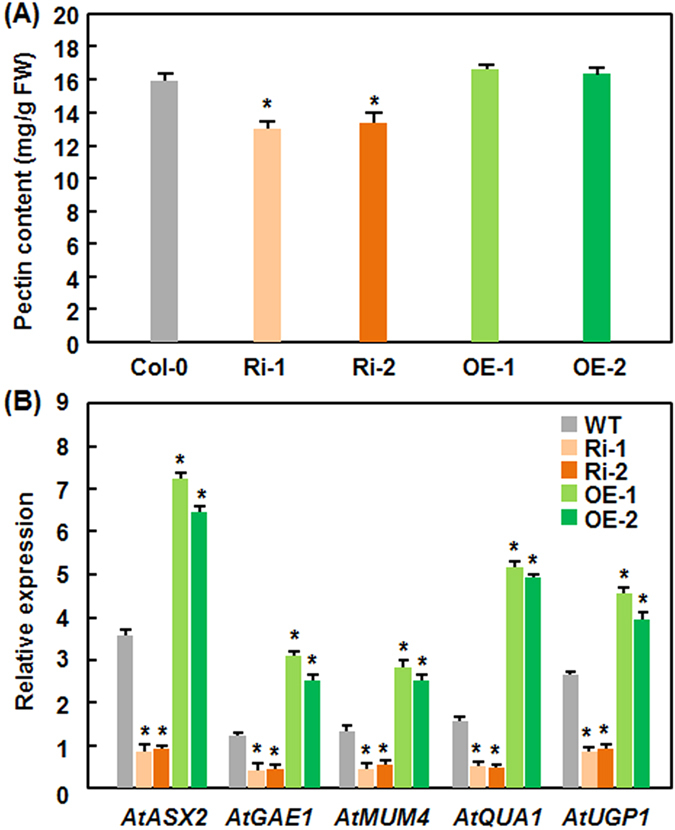
Altered pectin content and expression of pectin biosynthesis-related genes in AtERF014-OE and AtERF014-RNAi plants. (**A**) Pectin contents in WT, AtERF014-OE and AtERF014-RNAi plants. (**B**) Expression of pectin biosynthesis-related genes in WT, AtERF014-OE and AtERF014-RNAi plants. Data presented are the means ± standard deviation from three independent experiments and * above the columns indicate significant differences at *p* < 0.05 level between AtERF014-RNAi and WT plants.

It was previously reported that overexpression of *AtERF014* affected the expression of pectin biosynthetic genes and pectin deposition in cultivated Arabidopsis cells^42^. We thus analyzed and compared the pectin contents and expression levels of the pectin biosynthetic gene in WT, AtERF014-OE, and AtERF014-RNAi plants. As shown in Fig. 8A, the pectin contents in AtERF014-RNAi plants were measured to be 13.06 and 13.15 mg/g FW, respectively, significantly lower than that in WT (15.87 mg/g FW); however, the pectin contents in AtERF014-OE plants, measured as 16.57 and 16.29 mg/g FW, respectively, were comparable to that in WT. Accordingly, the expression levels of *AtASX2*, *AtGAE1*, *AtMUM4*, *AtQUA1* and *AtUGP1*, which are involved in pectin biosynthesis^50^,^51^,^52^,^53^,^54^,^55^, in AtERF014-OE plants were significantly higher but the levels in AtERF014-RNAi plants were markedly lower than those in WT (Fig. 8B). These results indicate that altered expression of *AtERF014* affects the pectin biosynthetic pathway, leading to altered pectin contents in AtERF014-OE and AtERF014-RNAi plants.

## Discussion

Fifteen ERF TFs are classified into group II^21^ and some of them have recently been functionally characterized. Overexpression of *AtERF012* (*DREB26*) resulted in deformed plants, indicating an involvement in developmental processes^56^. AtERF06 (AtRAP2.1) was found to function as a transcriptional repressor that plays a role in abiotic stress response^57^. AtERF011 (AtDEAR1) acts as an upstream regulator that mediates plant defense and freezing stress responses in Arabidopsis^40^ while AtERF018 seems to be involved in JA signaling pathway in response to wounding^58^. In the present study, we explored in detail the biological function of AtERF014 in immunity against different pathogens using AtERF014-OE and AtERF014-RNAi lines and found that AtERF014 acts as a dual regulator that differentially modulates immunity against *Pst* DC3000 and *B. cinerea*. These findings, together with a previous observation that AtERF014 is involved in pectin deposition in cultured Arabidopsis cells^43^, demonstrate a clear biological function of AtERF014 in biotic stress response.

Group II of the ERF subfamily consists of three subgroups, IIa, IIb, and IIc^21^. ERF TFs in this group all contain a common AP2/ERF domain at N-terminal and a conserved motif CMII-1 at C-terminals but also have several additional conserved motifs^21^. The six IIa members contain a CMII-2 motif that is similar to the EAR (ethylene response factor-associated amphiphilic repression) motif, which functions as a repression domain^22^,^59^. For instance, AtERF06 (AtRAP2.1) and AtERF011 (AtDEAR1) in subgroup IIa were experimentally demonstrated to be active transcriptional repressors that repress the transcription of some stress-responsive genes whose promoters harbor DRE (dehydration-responsive elements)/CRT (dehydration-responsive element) *cis*-elements^40^,^57^. By contrast, the members in subgroup IIb, except AtERF#015, have a CMII-3 motif at the C-terminal instead of the EAR-like CMII-2 motif 21. ERF TFs lacking an EAR motif are expected to act as transcriptional activators. We found that AtERF014 displayed transactivation activity in yeasts (Fig. 1D), implying that AtERF014 may be a transcriptional activator. Another member of subgroup IIb, AtERF012 (DREB26), was also found to have transactivation activity in yeast^56^. Thus, it seems likely that the group II ERFs have distinct biochemical activity and can act as transcriptional activators or repressors. Further transactivation assays in *in vivo* systems like Arabidopsis protoplasts or tobacco leaves are required to confirm the transactivation activity of AtERF014 and its closely related members in group II.

We showed in the present study that AtERF014 plays differential functions in immunity against *Pst* DC3000 and *B. cinerea*, demonstrating that AtERF014 is a dual regulator of immunity against these two pathogens. It is generally accepted that immune response against (hemi)biotrophic pathogens such as *Pst* DC3000 is modulated through the SA signaling while immune response against necrotrophic pathogens like *B. cinerea* is regulated by the JA/ET signaling^60^,^61^,^62^. Antagonistic interaction between the SA and JA/ET signaling pathways often occurs and allows plants to mount appropriate defense responses against different invading pathogens^60^,^62^,^63^,^64^. The expression of *AtERF014* was induced by *Pst* DC3000 and *B. cinerea* as well as by SA and MeJA (Fig. 1A–C). However, overexpression of *AtERF014* led to increased resistance to *Pst* DC3000 but decreased resistance to *B. cinerea*, whereas suppression of *AtERF014* resulted in decreased resistance to *Pst* DC3000 but increased resistance to *B. cinerea* (Figs 3 and 4), indicating that AtERF014 is a positive regulator of immunity to *Pst* DC3000 but a negative regulator of immunity to *B. cinerea*. This is similar to the functions of AtERF1, AtERF5 and AtERF6, which have opposite functions in immunity against *Pst* DC3000 and necrotrophic fungi including *B. cinerea* and *Alternaria brassicicola*^26^,^32^,^33^. The expression of SA-responsive and JA-responsive defense genes exhibited opposite patterns in AtERF014-OE and AtERF014-RNAi plants after infection of *Pst* DC3000 (Fig. 5C), indicating that AtERF014 is involved in modulating an antagonistic interaction between the SA and JA/ET signaling pathways, resulting in the expression of corresponding defense genes. It was previously shown that some of the B3 group members including ERF1, AtERF5, AtERF6, AtERF14 and ORA59 act as regulators of the JA/ET signaling pathway^26^,^27^,^30^,^31^,^32^,^42^. In contrast, only a few of ERFs, e.g. AtERF15 and AtERF5^33^,^35^, were shown to be involved in the SA signaling pathway. The involvement of AtERF014 in the SA signaling pathway can be further validated by the facts that the SA-induced *Pst* DC3000 resistance and SA-upregulated expression of defense genes were partially suppressed in AtERF014-RNAi plants (Fig. 7). The pathogen-induced ROS in AtERF014-OE plants (Fig. 5A,B) may function differentially in immunity against *Pst* DC3000 or *B. cinerea*. In the AtERF014-OE plants, the pathogen-induced ROS may largely contribute to signaling in immunity against *Pst* DC3000^65^, whereas this pathogen-induced ROS may benefit the infection by *B. cinerea*^66^, correlating with the altered resistance against these pathogens (Figs 3 and 4). Notably, *B. cinerea* did not induce significant accumulation of H_2_O_2_ but caused remarkable accumulation of superoxide anion in infected Arabidopsis plants (Fig. 5A,B). It was suggested that the kinetics of ROS accumulation is the better indicator of defense response than the absolute levels of ROS in many interactions^66^. Quantitative analysis of the kinetics of ROS accumulation in AtERF014-OE and AtERF014-RNAi plants after pathogen infection should clarify the involvement of H_2_O_2_ in AtERF014-mediated defense response against *B. cinerea*. In addition, the flg22-induced ROS burst and the expression of PTI marker genes *AtFRK1* and *AtWRKY53* were fostered in AtERF014-OE plants but was weakened in AtERF014-RNAi plants (Fig. 6), demonstrating that AtERF014 plays a role in innate immunity. AtERF5 and AtERF15 were also found to be involved in flg22- or chitin-induced PTI^33^,^35^.

Plant cell walls consist of three major components cellulose, hemicelluloses and pectins, and pectins make up approximately 50% of Arabidopsis leaf cell walls^67^. It was previously shown that overexpression of *AtERF014* coordinately activated the pectin biosynthetic pathway genes and increases the pectin content in cultured Arabidopsis cells^43^. We found in the present study that the pectin content in AtERF014-RNAi plants was significantly decreased due to the downregulated expression of pectin biosynthetic gene such as *AtASX2*, *AtGAE1*, *AtMUM4*, *AtQUA1* and *AtUGP1*^50^,^51^,^52^,^53^,^54^,^55^ (Fig. 8). Although overexpression of *AtERF014* upregulated the expression of these pectin biosynthetic genes, the pectin content in AtERF014-OE plants was comparable to that in WT (Fig. 8). This is contrary to the observation in cultured Arabidopsis cells, where overexpression of *AtERF014* led to increased pectin content^43^.

Plant cell walls act as preformed structural barriers against pathogen entry and modifications of cell walls can induce plant defense responses, which are able to reduce pathogen penetration and growth^68^. In the present study, we found that suppression of *AtERF014* resulted in reduced pectin content in AtERF014-RNAi plants as compared to WT (Fig. 8A); however, the contribution of the reduced pectin content to the resistance against *Pst* DC3000 and *B. cinerea* may differ (Figs 3 and 4). It seems reasonable that the reduced pectin content in AtERF014-RNAi plants may be responsible for the decreased resistance against *Pst* DC3000, as decrease in pectin content should weaken the cell wall strength and integrity and thereby make it more susceptible to *Pst* DC3000 infection. This is in agreement with a recent observation that mutation in *GAE6*, coding for a glucuronate 4-epimerase involved in the biosynthesis of pectin, impaired the immunity to *P. syringae* pv. *maculicola* ES4326^69^. However, the Arabidopsis *powdery mildew-resistant* (*pmr*) *5* and *pmr6* mutant plants, whose cell walls contain enriched pectin, are fully susceptible to *Pst* DC3000, as the WT plants^70^,^71^, indicating that increased pectin content in *pmr5* and *pmr6* plants is not responsible for the resistance to this bacterial pathogen. On the other hand, most of the necrotrophic fungal pathogens like *B. cinerea* secrete a variety of cell wall-degrading enzymes including polygalacturonases that can degrade pectin in cell walls during pathogenesis. *B. cinerea* was also shown to metabolize pectin and probably utilize pectin as a carbon source during *in planta* growth^72^,^73^. In this regard, reduced pectin content in AtERF014-RNAi plants (Fig. 8A) may contribute to increased resistance to *B. cinerea* (Fig. 4A), as the AtERF014-RNAi plants supported less *in planta* growth of *B. cinerea* in comparison to that in WT (Fig. 4B). This finding is different from the observation that the *gae1 gae6* plants, which are reduced in pectin content, impaired the resistance to specific *B. cinerea* isolates and allowed enhanced *in planta* growth of these specific isolates^69^. The AtERF014OE plants had similar pectin content to WT (Fig. 8A) but showed enhanced susceptibility to *B. cinerea* and *in planta* fungal growth (Fig. 3A,B), indicating that utilization of pectin as a carbon source may not contribute to immunity against *B. cinerea*. It is likely that the pectin content itself may not be a contributing factor that determines the immunity to pathogens; instead, the degree and pattern of pectin modification such as methylesterification are critical to immunity against *P. syringae*^74^,^75^,^76^ or *B. cinerea*^77^,^78^. It was previously shown that pectin fragments known as oligogalacturonides can trigger plant immune responses^69^,^79^,^80^. How AtERF014 regulates the biosynthesis of pectin and thereby the immunity against different pathogens need to be further investigated.

The involvement of ERFs in plant growth and development has been well-documented^81^. No morphological and developmental changes (Fig. 2) and altered expression of defense genes (Fig. 5C,D) were observed in the AtERF014-OE and AtERF014-RNAi plants, indicating that AtERF014 may not play a significant role in growth and development. This is in consistent with the observation that overexpression or suppression of *AtERF5*, *AtERF6* or *AtERF15* did not result in any distinguishable morphological and developmental changes to WT plants^33^,^35^,^82^.

## Conclusion

Our data presented in this study demonstrate that AtERF014 is a nucleus-localized transcriptional factor that acts as a dual regulator, which differentially modulates immunity against *Pst* DC3000 and *B. cinerea*, probably through regulating pectin biosynthesis pathway. However, how AtERF014 regulates plant immunity against *Pst* DC3000 and *B. cinerea* is largely unknown. This question can be explored by the characterization of *AtERF014*-mediated regulon and its direct target genes. Comparative RNA-seq analysis of the AtERF014-RNAi and WT plants and ChIP-seq analysis of transgenic palnts with expression of epitope tagged AtERF014, with or without pathogen infection, will not only lead to the identification of AtERF014-dependent differentially expressed regulon, but also provide information on the AtERF014 binding sites at genome-wide level. Data from these analyses will definitely be helpful to characterize the direct target genes, putative pathways and transcriptional network that are regulated by AtERF014 during immune response against pathogens.

## Methods

### Plant growth and treatments

Arabidopsis ecotype Columbia-0 (Col-0) was used for generation of transgenic lines and for phenotype assays. Plants were grown in a vermiculite: plant ash: perlite (6:2:1) mixture in a growth room at 24 °C with a cycle of 16 hr light/8 hr dark. For analysis of gene expression after pathogen infection, procedures for pathogen inoculation were described below and plants treated with similar volume of buffers were used as mock-inoculation controls. For analysis of gene expression after hormone treatment, SA and MeJA were dissloved in 0.1% ethanol to concentrations of 1 mM and 100 μM, respectively. Four-week-old plants were treated by spraying with SA or MeJA solutions or with 0.1% ethanol solution as mock-treated controls. The inoculated or treated plants were kept in sealed containers to maintain high humidity and leaf samples were collected at indicated time points.

### Generation and characterization of transgenic lines

The *AtERF014* cDNA was obtained by PCR amplification using primers AtERF014-orf-1F (5′-ATG GTG AAA ACA CTT CAA-3′) and AtERF014-orf-1R (5′-TCA GCA GAA GTT CCA TAA-3′) and cloned into pMD19-T vector. After confirmation by sequencing, the resulting plasmid pMD19-AtERF014 was used as templates for all experiments described below. For construction of overexpression vector, the *AtERF014* coding sequence was amplified with a pair of primers AtERF014-orf-2F (5′-GCG GAATTC ATG GTG AAA ACA CTT CAA AAG AC-3′, a *Bam*HI site underlined) and AtERF014-orf-2R (5′-AGA GGGCCC GTA TAA TGA AAG TAG AAT CAG CAG-3′, a *Sma*I site underlined) and inserted into pCAMBIA 99-1 under the control of CaMV 35S promoter. For construction of RNAi vector, a fragment was amplified with a pair of primers AtERF014-RNAi-F (5′-AGA TTAATTAA CCATGG AAC ACT TCA AAA GAC ACC AAA GAG A-3′, *Pac*I and *Nco*I sites underlined) and AtERF014-RNAi-R (5′-GCG GGATCC GGCGCGCC GAG TGC TGC GTC GTA GGC TCT AGC C-3′, *Eco*RI and *Asc*I sites underlined) and inserted into vector pGSA1165. Arabidopsis transformation was performed through the floral dip method^83^. Putative single-copy transgenic lines and homozygous lines were obtained by screening for a 3:1 segregation ratio of hygromycin-resistant (Hgr^R^) character and 100% Hgr^R^ phenotype in T2 and T3 generations on 1/2 MS medium supplemented with 50 μg/mL Hgr, respectively.

### Transactivation activity and subcellular localization assays

For transactivation activity assay, the *AtERF014* coding sequence was amplified by PCR with a pair of primers AtERF014-y-1F (5′-AGT GAATTC ATG GTG AAA ACA CTT CAA-3′, an *Eco*RI site underlined) and AtERF15-y-1R (5′-AGT GTCGAC TCA GCA GAA GTT CCA TAA-3′, a *Sal*I site underlined) and inserted into pBD vector between *Eco*RI and *Sal*I sites. The resulting plasmid pBD-AtERF014, pBD-GAL4 (as a positive control) and pBD empty vector (as a negative control) were introduced into yeast strain AH109 and the yeast transformants were plated and grown on SD/Trp^–^ medium at 30 °C for 3 days. Transactivation activity was assessed according to the growth status on SD/Trp^–^ plate and the production of blue pigment after addition of X-α-gal. For subcellular localization assay, the *AtERF014* coding sequence was obtained by PCR with a pair of primers AtERF014-s-1F (5′-GCG TCTAGA ATG GTG AAA ACA CTT CAA-3′, a *Xba*I site underlined) and AtERF15-s-1R (5′-ATA CCCGGG TCA GCA GAA GTT CCA TAA-3′, a *Sma*I site underlined), and inserted into vector pFGC-EGFP between *Xba*I and *Sma*I sites. The recombinant plasmid pFGC-GFP-AtERF014 and the empty vector pFGC-EGFP were transformed into *Agrobacterium tumefaciens* strain GV3101. Agrobacteria harboring pFGC-GFP-AtERF014 or pFGC-EGFP were infiltrated into leaves of *N. benthamiana* plants expressing a red nuclear marker protein RFP–H2B^44^ and the agroinfiltrated leaves were collected 24 hr later. Fluorescence signals were excited at 488 nm and detected under a confocal laser scanning microscope (LSM 510 Meta, Zeiss, Oberkochen, Germany) using a 500–530 nm emission filter.

### Disease assays and measurement of *in planta* pathogen growth

*Pst* DC3000 was grown in King’s B (KB) broth and collected by centrifugation, followed by re-suspending in 10 mM MgCl_2_ solution to OD_600_ = 0.002. The bacterial inoculation was performed by hand infiltration using 1-ml syringes without needle into rosette leaves and the inoculated plants were kept in sealed containers to facilitate disease development under high humidity. For quantification of *in planta* bacterial growth, leaf discs from inoculated leaves were collected at different time points and homogenized in 10 mM MgCl_2_. After a series of gradient dilutions, the homogenate was plated on KB plates supplemented with 25 μg/mL rifampicin and bacterial colonies were counted at 2 days after incubation at 28 °C. *B. cinerea* strain BO5.10 (provided by Dr. Tesfaye Mengiste, Purdue University, USA) was grown on 2 × V8 agar^84^ and spores were collected, resuspended in 1% maltose buffer to a final concentration of 2 × 10^5^ spores/mL. Plant inoculation, measurement of chlorophyll content and recoding of dead plants were followed by previously described methods^84^,^85^. Measurement of *in planta* fungal growth was performed by analyzing the transcript level of *B. cinerea BcActinA* gene and comparing with the transcript level of an Arabidopsis actin gene as an internal control according to a reported protocol^35^,^85^.

### Measurement of ROS burst and *in situ* detection of ROS accumulation

Measurement of ROS burst was carried out using a luminol-based luminescence method^86^. Briefly, 4-mm leaf discs were floated in 200 μL H_2_O overnight and then transferred into 100 μL 100 nM flg22 solution containing 34 μg/ml luminol (Sigma, St. Louis, MO, USA) and 20 μg horseradish peroxidase (Sigma, St. Louis, MO, USA) or in solution without luminol as controls. Luminescence was recorded at a 2 min interval over 20 min using a Synergy HT plate reader (Biotek Instruments Inc. Winooski, VT, USA). *In situ* detection of superoxide anion and H_2_O_2_ was carried out by NBT and DAB staining, respectively^45^,^46^. Accumulation of ROS in stained leaves was visualized by a digital camera.

### Measurement of pectin content

Pectin content was determined using a Pectin Measurement kit (Keming Biotechnology Co., Suzhou, China) according to the manufacturer’s instruction. Briefly, 100 mg of leaf tissues was immerged into 1 mL sterile water and then ground to homogenate. After centrifugation at 8000 × g at 4 °C for 10 min, the supernatant was used for measurement of pectin.

### qRT-PCR analysis of gene expression

Total RNA was extracted by Trizol reagent (Invitrogen, Shanghai, China) according to the manufacturer’s instruction. First-strand cDNA was synthesized from 1 μg total RNA using PrimeScript RT kit (TaKaRa, Dalian, China) following the supplier’s recommendation. Quantitative RT-PCR was prepared using SYBR Premix Ex Taq Kit (TaKaRa, Dalian, China) according to the manufacturer’s instruction and run on a CFX96 real-time PCR system (BioRad, Hercules, CA, USA). Gene-specific primers used in qRT-PCR were as follows: AtERF014-q-1F, 5′-CTG CCA ACA CAG ATC CTT CCT CAT-3′; AtERF014-q-1R, 5′-AAA TAC GGC GCG GAG ACT CCA TTA-3′; AtPR1-q-F, 5′-TCG TCT TTG TAG CTC TTG TAG GTG-3′; AtPR1-q-R, 5′-TAG ATT CTC GTA ATC TCA GCT CT-3′; AtPR3-q-F, 5′-GGC CAG ACT TCC CAT GAA AC-3′; AtPR3-q-R, 5′-CTT GAA ACA GTA GCC CCA TGA A-3′; AtPR5-q-F, 5′-ATG GCA AAT ATC TCC AGT ATT CAC A-3′; AtPR5-q-R, 5′-ATG TCG GGG CAA GCC GCG TTG AGG-3′; AtPDF1.2-q-F, 5′-GCTA AGT TTG CTT CCA TCA TCA CCC TT-3; AtPDF1.2-q-R, 5′-AAC ATG GGA CGT AAC AGA TAC ACTTGT G-3′; AtNPR1-q-1F, 5′-CAC TAT GGC GGT TGA ATG TA-3′; AtNPR1-q-1R, 5′-GGG AGG AAC ATC TCT AGG AA-3′; AtASX2-q-1F, 5′-GGG TGC TGA GAA TGG ACT CG-3′; AtASX2-q-1R, 5′-CTC CCG TCG CAG AAG ATT G-3′; AtGAE1-q-1F, 5′-GAA GAT GAG CTG TTT CCG TCA-3′; AtGAE1-q-1R, 5′-AAG AAG CGG TGA GAG CGA TG-3′; AtMUM4-q-1F, 5′-TGA GGG TTC GGG TAT AGG TTT C-3′; AtMUM4-q-1R, 5′-AGG TCT GAG GAG ATT GGC ATC-3′; AtQUA1-q-1F, 5′-TCT CTC CTT CTT CTT CGC TTC A-3′; AtQUA1-q-1R, 5′-GAT CTG AAT CAA CCG TGG CTT A-3′; AtUGP1-q-1F, 5′-CGT TCG ATG GCC TTA CTG AGA-3′; AtUGP1-q-1R, 5′-GAT TCT TGG TCT CGG CAA CAT-3′; BcActinA-q-1F, 5′-ACT CAT ATG TTG GAG ATG AAG CGC AA-3′; BcActinA-1R, 5′-AAT GTT ACC ATA CAA ATC CTT ACG GAC A-3′; AtActin-q-1F, 5′-GGC GAT GAA GCT CAA TCC AAA CG-3′; AtActin-q-1R, 5′-GGT CAC GAC CAG CAA GAT CAA GAC G-3′. An Arabidopsis Actin gene was used as an internal control to normalize the data and relative expression levels of genes of interest were calculated using the 2^−ΔΔCT^ method.

### Statistical analysis

All experiments were repeated independently for at least three times and data were subjected to statistical analysis using the Student’s *t-*test at *p* = 0.05 level.

## Additional Information

**How to cite this article**: Zhang, H. *et al*. Arabidopsis AtERF014 acts as a dual regulator that differentially modulates immunity against *Pseudomonas syringae* pv. *tomato* and *Botrytis cinerea*. *Sci. Rep.* **6**, 30251; doi: 10.1038/srep30251 (2016).
